# Higher serum galactose-deficient immunoglobulin A1 concentration is associated with stronger mesangial cellular inflammatory response and more severe histologic findings in immunoglobulin A nephropathy

**DOI:** 10.1093/ckj/sfy068

**Published:** 2018-08-03

**Authors:** Celine Nguyen, Katrin König, Frederick W K Tam, Helmut Hopfer, Karen Molyneux, Francoise-Isabelle Binet, Min Jeong Kim

**Affiliations:** 1 Department of Biomedicine, University of Basel, Basel, Switzerland; 2 Clinic for Transplantation Immunology and Nephrology, University Hospital Basel, Basel, Switzerland; 3 Renal and Transplant Centre, Renal and Vascular Inflammation Section, Department of Medicine, Hammersmith Hospital, Imperial College London, London, UK; 4 Institute for Pathology, University Hospital Basel, Basel, Switzerland; 5 Department of Infection, Immunity & Inflammation, University of Leicester, Leicester, UK; 6 Clinic for Nephrology and Transplantation Medicine, Kantonsspital St. Gallen, St. Gallen, Switzerland

**Keywords:** Gd-IgA1, IgA nephropathy, MCP-1, mesangial cellular inflammatory response, Oxford classification MEST

## Abstract

**Background:**

Galactose-deficient immunoglobulin A1 (Gd-IgA1) is known to play a key role in the pathogenesis of IgA nephropathy (IgAN). We aimed to evaluate whether serum Gd-IgA1 is associated with *in vitro* activation of mesangial cells in individual patients and how this affects the clinical and histologic parameters.

**Methods:**

Serum samples and clinical and histologic data were collected in the University Hospital Basel and Hammersmith Hospital, London. Serum levels of IgA1 and Gd-IgA1 were measured by enzyme-linked immunosorbent assay (ELISA) and lectin-binding assay using lectin *Helix aspersa* (HA). Primary human mesangial cells were stimulated with IgA1 isolated from serum from individual patients and the concentrations of monocyte chemoattractant protein-1 and interleukin-6 were measured in cell culture supernatant by ELISA.

**Results:**

Thirty-three patients were enrolled. A significant correlation was observed between serum Gd-IgA1 levels and the concentration of MCP-1 in the culture supernatant in individual patients (Spearman *r* = 0.5969, P = 0.0002). There was no significant correlation between serum Gd-IgA1 levels and proteinuria or estimated glomerular filtration rate at diagnosis. However, the serum Gd-IgA1 level was significantly higher in patients with segmental glomerulosclerosis (S0 versus S1, P = 0.0245) and tubular atrophy/interstitial fibrosis (T0 versus T1 and T2, P = 0.0336; T0 versus T2, P = 0.0225).

**Conclusions:**

Higher serum Gd-IgA1 concentration is associated with stronger mesangial cell inflammatory response with production of a greater amount of MCP-1 *in vitro.* This in turn is associated with severe histologic changes. The disease progression with worse renal outcome in patients with higher serum Gd-IgA1 may be therefore mediated by more pronounced mesangial cell inflammatory response leading to more severe histologic changes.

## INTRODUCTION

It has been >15 years since an excess of galactose-deficient immunoglobulin A1 (Gd-IgA1) was found to be present both in the serum and in the glomerular immune deposits of patients with IgA nephropathy (IgAN) [1]. IgA1 is unusual in that it has a heavily *O*-glycosylated hinge region between the C_H_1 and C_H_2 domains of the α heavy chain [2, 3]. The presence of galactose-deficient IgA1 *O*-glycoforms favours formation of circulating immune complexes (ICs), which are prone to deposition in the glomerular mesangium [1, 4–6]. Although the exact mechanism of mesangial IgA1 deposition is not yet satisfactorily defined, these IgA ICs trigger mesangial cell activation, proliferation and apoptosis [7, 8]. Activated mesangial cells release various pro-inflammatory, pro-proliferative and pro-fibrogenic mediators and the subsequent inflammation, cellular proliferation and synthesis of extracellular matrix lead to progression of IgAN [9–14]. 

The clinical presentation and disease progression of IgAN are highly variable and it is therefore strongly desirable to recognize patients who are most likely to progress to chronic kidney disease and end-stage renal disease (ESRD) and to derive the most benefit from therapy, especially immunosuppressive therapy. Therapy recommendations have been based on clinical findings at first presentation or at the time of renal biopsy, such as the degree of proteinuria, reduced glomerular filtration rate (GFR) and the presence of hypertension [15]. Certain histologic features on renal biopsy, such as mesangial hypercellularity, segmental glomerulosclerosis, interstitial fibrosis/tubular atrophy and crescents also predict a worse outcome [16, 17].

Many studies have so far shown that the level of serum Gd-IgA1 is elevated in patients with IgAN compared with healthy controls [18–20]. These studies have also shown that the level of serum Gd-IgA1 at the time of renal biopsy significantly correlates with disease progression [19, 20]. In another study, the level of Gd-IgA1 was associated with more severe histologic findings [21]. Hoever, this parameter however, did not significantly correlate with the classical risk parameters, such as proteinuria or estimated glomerular filtration rate (eGFR), raising the question of if the classical parameters are sufficient for risk assessment [19, 20]. We know from our own data and others that Gd-IgA1 plays a key role in the activation of mesangial cells and the crosstalk between the different cell types present in glomeruli in the pathogenesis of IgAN [22–25]. However, it has not been investigated so far whether the level of Gd-IgA1 in individual patients correlates with the activation of mesangial cells. Different levels of Gd-IgA1 in individual patients may lead to a variable inflammatory response of mesangial cells. This may suggest that patients with high Gd-IgA1 have stronger inflammatory responses and therefore greater potential for disease progression. We therefore investigated whether the serum level of Gd-IgA1 correlates with mesangial inflammatory response to IgA1 isolates from patients and also clinical and histologic findings in individual patients.

## MATERIALS AND METHODS

### Study design

We performed a multicentre cross-sectional study with prospective enrolment of patients with newly diagnosed IgAN.

### Patients

Patients ≥18 years of age attending University Hospital Basel (BS) and Imperial College Renal and Transplant Centre, Hammersmith Hospital, London, UK (HS) with newly diagnosed primary IgAN by kidney biopsy were enrolled in the study. Patients with concomitant diagnosis in the kidney biopsy or a secondary form of IgAN were excluded from the study. Patients requiring renal replacement therapy at the time of diagnosis were also excluded. All patients gave written informed consent for the use of their data and samples for research. The study was approved by the Research Ethics Committee of the Canton of Basel (no. 301/12), the National Research Ethics Service Committee London–West London (REC reference 04/Q0406/25) and the Imperial College Healthcare National Health Service Trust.

### Data collection and kidney biopsy

Clinical and epidemiological data and laboratory parameters [serum creatinine, eGFR calculated using Chronic Kidney Disease Epidemiology Collaboration formula, spot urine protein:creatinine ratio (PCR) and urine sediment] were collected at the time of diagnosis.

Kidney biopsy was evaluated by independent renal pathologists in BS and HS. The mesangial hypercellularity (M), endocapillary hypercellularity (E), segmental glomerulosclerosis (S), tubular atrophy and interstitial fibrosis (T)-C system of Oxford classification was used for scoring [16, 17].

### Sample collection and measurements of serum total IgA1 and Gd-IgA1

Serum samples were collected at enrolment and stored in a –80°C freezer until further assay or isolation of IgA1. Serum IgA1 was quantified by specific ELISA. In brief, 96-well immunoplates were coated with rabbit anti-human antibodies to IgA (A0262; Dako, Carpenteria, CA, USA), followed by a blocking step with 2% bovine serum albumin (BSA) in phosphate-buffered saline (PBS). A total of 50 μL aliquots of standard and test serum samples were applied to duplicate wells. Standard curves were set up on each plate, using a National Institute for Biological Standards and Control serum standard (no. 67/099) ranging from 863 to 1.7 ng/mL for IgA1. Serum samples were diluted in PBS at 1:20 000. After overnight incubation, secondary antibodies to human IgA1 [sheep anti-human IgA1 (binding site)] were added for 2 h incubation. For the development, horseradish peroxidase (HRP)-conjugated anti-sheep/goat immunoglobulin antibody (binding site) was first applied for 1.5 h incubation, followed by o-phenylenediamine (OPD)/(hydrogen peroxide H_2_O_2_). The results were read as absorbance at 492 nm.

Levels of Gd-IgA1 were measured by lectin-binding assay using N-acetylgalactosamine (GalNAc)-specific lectin from the *Helix aspersa* (HA) using a previously described ELISA method [1]. HA recognizes terminal *O*-linked GalNAc and samples with lower terminal galactosylation and sialylation show higher HA binding. Briefly, IgA was captured on 96-well immunoplates, coated overnight at 4°C with 10 μg/mL anti-human IgA antibody (A0262; Dako), washed and blocked with 2% BSA in PBS. Serum samples, diluted 1:100 in PBS, were applied to the plates (50 μL/well), in duplicate, and incubated overnight at 4°C. At this dilution, all wells on the plate were completely saturated with IgA1, which was confirmed by IgA1-specific ELISA. This enables an equivalent amount of total IgA1 from each sample to be tested by the assay. Gd-IgA1 was detected by incubation for 90 min with biotinylated HA (L8764: Sigma-Aldrich, St. Louis, MO, USA) followed by HRP-conjugated avidin (DY 998). The reaction was developed with OPD/H_2_O_2_ substrate and the results read as absorbance at 492 nm. To create an IgA1-HA binding standard curve, three patients with the highest IgA1-HA binding and three patients/healthy subjects with the lowest IgA1-HA binding from the Leicester IgA cohort were selected. Equal amounts of sera from the three high IgA1-HA binders were pooled to create the high standard and equal amounts of sera from the three low IgA1-HA binders were pooled to create the low standard. The high and low pooled sera standards were then serially combined to create 11 different standard serums with arbitrary units (AUs) of IgA1-HA binding ranging from 110 to 10. All standards were diluted 1:100 in PBS before use. The results were expressed as AUs. The intra- and interassay variations were <10%. For the validation of results, the measurement was repeated independently at the University of Leicester by adding a desialylation process using neuraminidase prior to the lectin binding step.

### Isolation of IgA1 from the serum sample

Serum from each patient was precipitated with saturated ammonium sulphate solution in 0.175 mol/L Tris-HCl (pH 7.5). The obtained fraction was incubated with Jacalin Agarose (Vector Laboratories, Burlingame, CA, USA) and IgA1 eluted from the Jacalin with 1 mol/L D-galactose in Tris-HCl. Buffer was changed using PD-10 desalting columns, the total volume reduced to 20% of the original volume using concentrator and the purified IgA1 samples stored at −80**°**C until use. The concentrations of purified IgA1 were measured by IgA1-specific ELISA.

### Stimulation of human mesangial cells (HMCs) with isolated IgA1 from individual patient

HMCs were purchased at passage number 3 (Lonza, Walkersville, MD, USA). Cells from the same donor were used for the *in vitro* experiments. HMCs were grown in mesangial cell basal medium supplemented with 5% foetal calf serum (FCS), gentamicin (30 ug/mL) and amphotericin B (15 ng/mL). Quiescent HMCs in Roswell Park Memorial Institute 1640 media containing 0.5% FCS, grown at passages 5–7, were stimulated with IgA1 isolated from each patient at 100 μg/mL. For standardization of the results, a negative control (only cell culture media) and a calibration positive control (pooled IgA1 from 33 patients) were used. Cell culture supernatant was collected after 24 h stimulation and pro-inflammatory cytokines monocyte chemoattractant protein-1 (MCP-1) and interleukin-6 (IL-6) were measured by ELISA using matched paired antibodies (R&D Systems, Minneapolis, MN, USA). The results were expressed as AUs in reference to calibration positive controls.

### Statistical analysis

For descriptive purposes, continuous variables with normal distribution are reported as mean ± SD. Skewed continuous variables are presented as median with interquartile range (IQR). Associations between continuous parameters were assessed using Spearman’s correlation coefficient and linear regression analysis. Differences between groups were analysed by Mann–Whitney U test. All hypothesis tests were two-sided and the significance level was set to 5%. Statistical analyses were performed using Prism 7.0 (GraphPad Software, La Jolla, CA, USA).

## RESULTS

### Baseline clinical data

Thirty-three patients with newly diagnosed primary IgAN were enrolled in the study at the University Hospital Basel and Hammersmith Hospital, London. Baseline characteristics of the patients are detailed in Table 1. All subjects had biopsy-proven IgAN. Twenty-one patients (64%) had an eGFR <60 mL/min/1.73 m^2^ and 20 patients showed proteinuria >1 g/day at the time of enrolment. Sixteen patients (53%) had hypertension and nine patients were treated with either angiotensin-converting enzyme inhibitor or angiotensin receptor blocker. Twenty-three patients showed mesangial hypercellularity (>50% of glomeruli) and segmental glomerulosclerosis, whereas endocapillary hypercellulartiy was seen in only six patients.

**Table 1.** Patient characteristics

Characteristics	Values
Age (year), median (IQR)	43 (20–85)
Gender (male), *n* (%)	24 (73)
Ethnicity (Caucasian/Asian/African), *n*	25/7/1
BMI (kg/m^2^), mean ± SD	25 ± 5
SBP (mmHg), mean ± SD	142 ± 18
DBP (mmHg), mean ± SD	87 ± 13
Hypertension, *n* (%)	16 (53)
History of macroscopic haematuria, *n* (%)	5 (17)
Microscopic haematuria, *n* (%)	30 (91)
Spot urine PCR (mg/mmol), median (IQR)	120 (12–548)
<50, *n*	5
50–99, *n*	8
100–299, *n*	12
>300, *n*	8
eGFR (mL/min per 1.73 m^2^), mean ± SD	55 ± 31
CKD Stages 1–5, *n*	5/7/14/4/3
Oxford classification score MEST-C, *n*	
M 0/1	10/23
E 0/1	27/6
S 0/1	10/23
T 0/1/2	16/10/7
C 0/1/2	17/14/2
Immunosuppression after diagnosis, *n*	11
ACE inhibitor or ARB (prior to/after diagnosis)	
ACE inhibitor	3/15
ARB	5/7
Dual blockade	1/3

Results are presented as mean ± SD or median with range. BMI, body mass index; SBP, systolic blood pressure; DBP, diastolic blood pressure; ACE, angiotensin-converting enzyme; ARB, angiotensin receptor blocker; M, mesangial hypercellularity; E, endocapillary hypercellularity; S, segmental glomerulosclerosis; T, tubular atrophy/interstitial fibrosis.

### Serum Gd-IgA1 concentration

**FIGURE 1:**
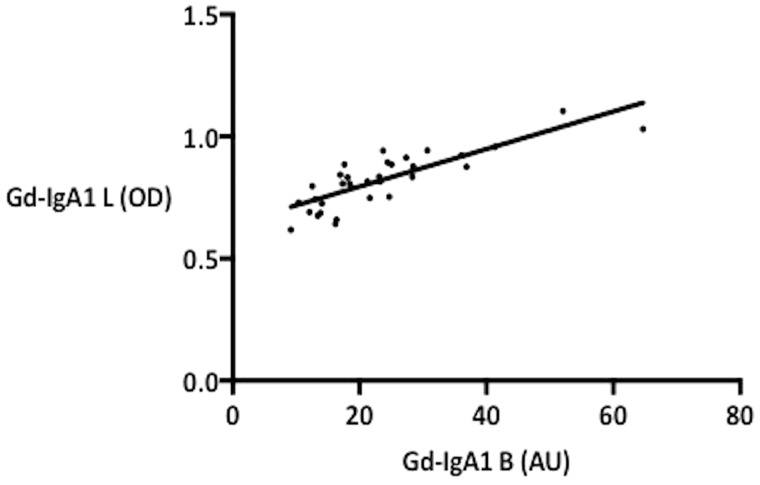
Correlation of Gd-IgA1 values from two independent measurements.

The measurement of Gd-IgA1 concentration was performed both at the University of Basel and University of Leicester. The results from both laboratories showed positive correlation, with Spearman *r* = 0.8419 (P < 0.0001) (Figure 1). For further analyses, the results from the University of Basel were used.

### Correlation of serum Gd-IgA1 concentration with cytokine MCP-1 produced by HMCs *in vitro* and clinical and histologic parameters

**FIGURE 2:**
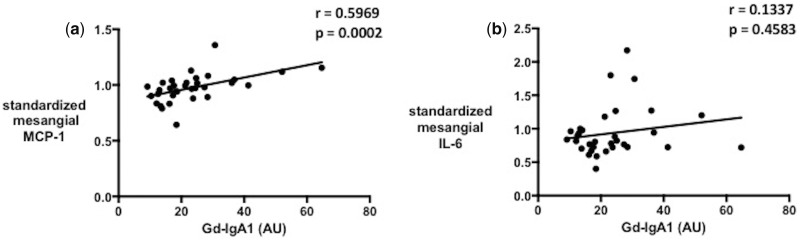
Correlation of serum Gd-IgA1 with (**a**) MCP-1 and (**b**) IL-6 from HMCs treated with IgA1 isolates from patients.

**FIGURE 3:**
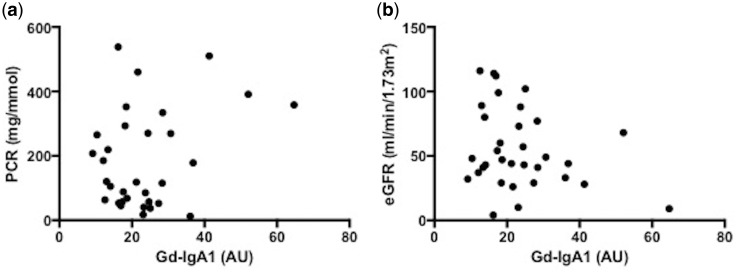
Correlation of serum Gd-IgA1 with proteinuria: (**a**) PCR and (**b**) eGFR at the time of diagnosis.

A significant correlation was observed between the serum Gd-IgA1 level and the MCP-1 concentration in the cell culture supernatant in individual patients (Spearman *r* = 0.5969, P = 0.0002) but not between serum Gd-IgA1 and the IL-6 concentration (Figure 2a and b and Supplementary data, Figure S1). The serum level of Gd-IgA1 did not correlate with clinical parameters such as proteinuria or eGFR (Figure 3a and b). There were also no significant correlations of MCP-1 or IL-6 concentration in the culture supernatant with eGFR or proteinuria.

**FIGURE 4:**
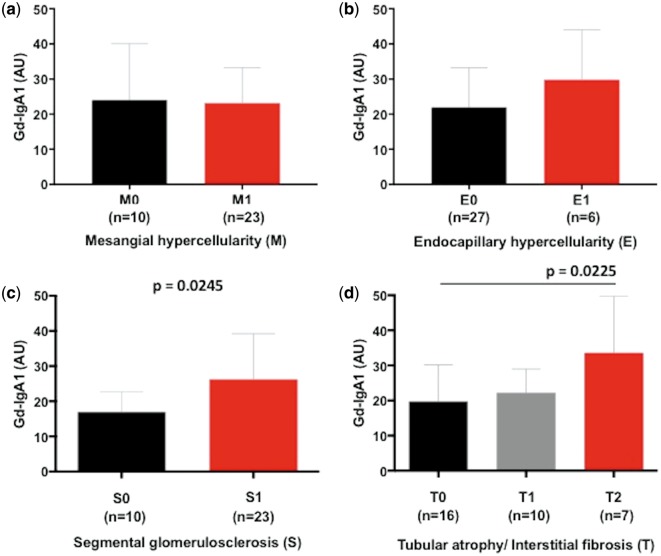
Serum Gd-IgA1 concentration according to Oxford classification MEST score, (**a**) M, (**b**) E, (**c**) S and (**d**) T.

We then looked at the association of serum Gd-IgA1 concentration with histologic parameters of the Oxford classification: mesangial hypercellularity (M), endocapillary hypercellularity (E), segmental glomerulosclerosis (S), tubular atrophy/interstitial fibrosis (T) and crescents (C). There was no significant difference in serum Gd-IgA1 concentration between M0 and M1, and this was also true between E0 and E1 (Figure 4a and b). However, patients with a higher serum level of Gd-IgA1 showed a significantly higher S score (S0 versus S1, P = 0.0245) and T score (T0 versus T1 and T2, P = 0.0336; T0 versus T2, P = 0.0225) (Figure 4c and d). Patients with the highest scores in S and T (S1 and T2) had significantly higher serum levels of Gd-IgA1 compared with the remaining patients (P = 0.0069).

**FIGURE 5:**
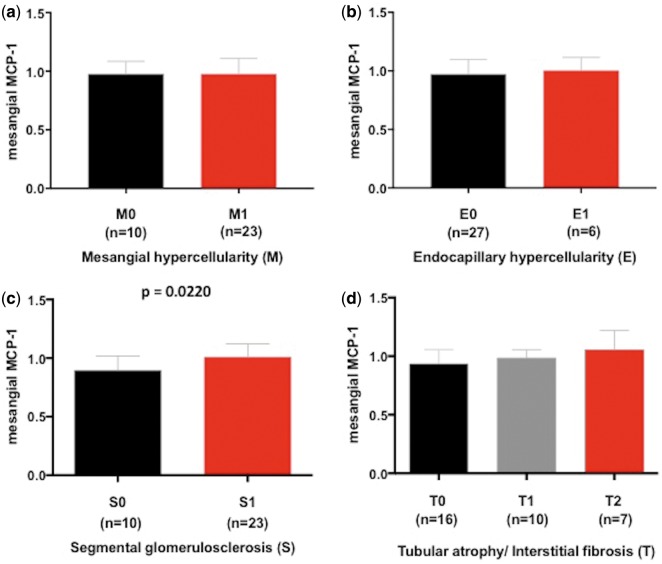
MCP-1 concentration in cell culture supernatant according to Oxford classification MEST score: (**a**) M, (**b**) E, (**c**) S and (**d**) T.

The MCP-1 concentration in the culture supernatant was not significantly different between M0 and M1 or E0 and E1 (Figure 5a and b). However, there was a significant difference in MCP-1 concentration in the culture supernatant between S0 and S1 (P = 0.0220). In addition, there was a trend towards a higher MCP-1 concentration in patients with higher T scores (Figure 5c and d).

We also evaluated the level of serum Gd-IgA1 and MCP-1 concentration in the culture supernatant according to the C score (Supplementary data, Figure S2a and b). Score C2 was observed in only two patients. Even though there was a trend of higher Gd-IgA1 levels in patients with higher C scores, there was no statistically significant difference between patients with different C scores. There was also no significant difference in MCP-1 concentration in patients with different C scores.

## DISCUSSION

In this study we examined whether individually isolated IgA1 from patients with different concentrations of serum Gd-IgA1 induces different degrees of inflammatory response on HMCs. We were able to demonstrate that HMCs treated with IgA1 isolates from patients with higher serum Gd-IgA1 concentrations produce greater amounts of MCP-1. Since the isolation of IgA1 and the stimulation of mesangial cells were performed in an identical manner, the difference in the mesangial cell responses has to be explained by different concentrations of Gd-IgA1 and/or IgA1 ICs in the IgA1 isolates. For the measurement of serum Gd-IgA1 concentration, there is so far no standardized method, and several methods from different laboratories have been reported in the literature [1, 26]. We measured the serum Gd-IgA1 concentration by lectin-binding assay using GalNAc-specific lectin from the HAs. To verify our results, the Gd-IgA1 concentration was measured independently at the University of Leicester, whereby the measurement method was slightly modified by the addition of a desialylation process using neuraminidase prior to the lectin binding step. The results from two independent laboratories showed excellent correlations, suggesting the reliability of our results.

In our previous work we showed that HMCs treated with pooled IgA1 isolates from IgAN patients produce both MCP-1 and IL-6, and that their production is reduced by the same inhibiting treatment [22]. In this study, whereas there was a positive correlation of MCP-1 concentration in the mesangial cell culture supernatant with the patient’s serum Gd-IgA1 level, there was no significant correlation between IL-6 and serum Gd-IgA1. It remains speculative whether there had been a significant correlation of IL-6 with a greater sample number.

The serum levels of Gd-IgA1 did not correlate with clinical findings such as proteinuria or eGFR at the time of IgAN diagnosis, although there was some trend for lower eGFR and higher proteinuria with increasing Gd-IgA1 concentration. This finding is consistent with previous reports from Chinese and Italian IgAN populations [19, 20]. They and others demonstrated that higher Gd-IgA1 concentration is associated with disease progression [18].

In terms of histologic parameters, the serum Gd-IgA1 concentration was significantly higher in patients with a higher Oxford classification score for segmental glomerulosclerosis or tubular atrophy/interstitial fibrosis. Correspondingly the MCP-1 production by HMCs treated with IgA1 isolates was higher in patients with a higher score for segmental glomerulosclerosis. There was also a trend for increasing MCP-1 production with increasing score for tubular atrophy/interstitial fibrosis, even though the difference was not statistically significant. A previous report from a Chinese population showed higher Gd-IgA1 concentrations in patients with more severe histologic phenotypes [21]. Many validation studies of the Oxford classification have shown that S and T scores predict disease progression and worse renal outcome [27–29]. Our results suggest that rapid disease progression and worse renal outcome in patients with higher S and T scores may be mediated by a more severe inflammatory response of mesangial cells. On the other hand, a more severe mesangial cell inflammatory response seems to be associated with higher serum Gd-IgA1 concentrations. In a recent report there was significant improvement in the prediction of renal outcome by adding Oxford classification scores to clinical data, such as proteinuria, eGFR and mean arterial blood pressure at biopsy [30]. The addition of serum Gd-IgA1 concentration at biopsy might lead to a further improvement in renal outcome prediction. Interestingly, we did not see any significant difference in serum Gd-IgA1 between M0 and M1, although IgA1 isolates from patients with higher serum Gd-IgA1 induced a stronger inflammatory response of mesangial cells. This finding suggest that there is another pathogenic factor besides serum Gd-IgA1 involved in mesangial cell proliferation.

To our knowledge, our study is the first to examine HMC response *in vitro* to individually isolated IgA1 from IgAN patients. The small number of included patients in the study is certainly a substantial limitation. There were also only a few patients with a positive E score (endocapillary hypercellularity), which makes it difficult to examine the relevance of serum Gd-IgA1 concentration for this histologic finding. However, there are strengths in our study, namely that this is the first study showing that different serum Gd-IgA1 concentrations in individual patients are associated with variable degrees of mesangial cell response *in vitro*. Also, we were able to show the reproducibility of Gd-IgA1 data by measurement in two independent laboratories.

In conclusion, different serum Gd-IgA1 concentrations in individual patients are associated with a variable degree of mesangial cell inflammatory response as measured by the production of MCP-1 *in vitro.* This response is associated with severe histologic changes that are known to be associated with disease progression and more severe renal outcome. Our results therefore support the postulated role of Gd-IgA1 in the pathogenesis of IgAN. Disease progression with worse renal outcome in patients with higher serum Gd-IgA1 concentrations may be mediated by a more pronounced mesangial cell response, leading to more severe histologic changes. The addition of serum Gd-IgA1 concentration to the Oxford classification score and clinical parameters may further improve the prediction of renal outcome in IgAN patients.

## Supplementary Material

Supplementary Data
