# Supplementary material for: Higher serum galactose-deficient immunoglobulin A1 concentration is associated with stronger mesangial cellular inflammatory response and more severe histologic findings in immunoglobulin A nephropathy
Source: Clin Kidney J. 2018 Aug 3;12(2):232–8. doi: 10.1093/ckj/sfy068 (PMC6452211; doi:10.1093/ckj/sfy068)
Supplement: Supplementary Data [file sfy068_spplementary_data.pdf]

Supplementary figure 1

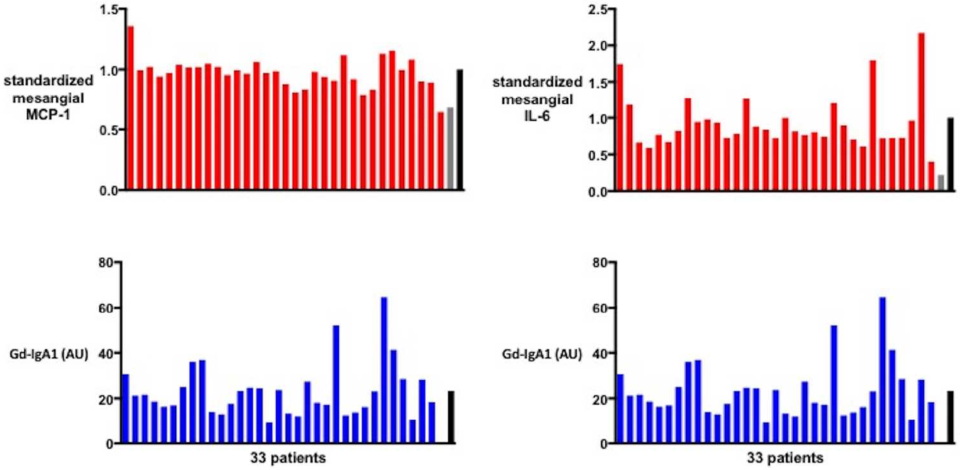

Supplementary Figure 1. Serum Gd-IgA1 and mesangial production of MCP-1 and IL-6. Grey bar represents the negative control and black bar the calibration positive control.

1058x793mm (72 x 72 DPI)

Supplementary figure 2

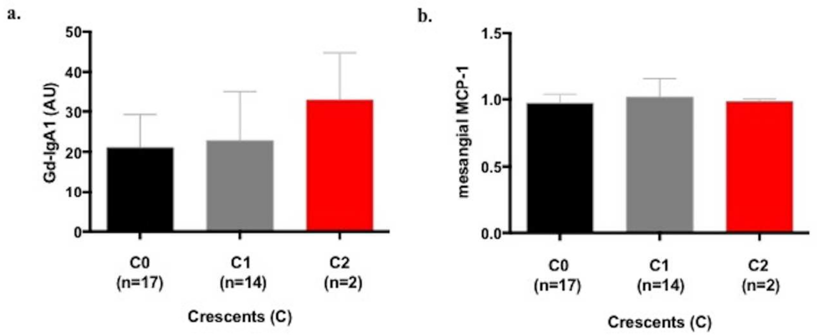

Supplementary Figure 2. Serum Gd-IgA1 and MCP-1 concentration in cell culture supernatant according to Oxford classification C score, Gd-IgA1 (a) & mesangial MCP-1 (b)

1058x793mm (72 x 72 DPI)
